# Diseases of the Ear

**Published:** 1895-09

**Authors:** 


*Diseases of the Ear.* By A. MARMADUKE SHEILD, M.B. Pp. xii., 266. London: CASSELL AND COMPANY, LIMITED. 1895.

This is an excellent text-book for students, written by a general surgeon who has done good work in other departments of surgery. It reminds us of the curious fact that the greatest advances in aural surgery have been made, in this country at least, by surgeons who are not merely specialists. We need only mention the names of Macewen, Barker, Hulke, Horsley, and Lane, as some of the foremost workers in this department of science; and now Mr. Sheild shows in the book before us how greatly a knowledge of general surgery facilitates a comprehensive grasp of this special department.

The book opens with a brief account of the anatomy and physiology of the ear, followed by a useful chapter on the examination of the ear. Affections of the auricle and external auditory canal are briefly dealt with, and a fairly full account of the diseases of the middle ear follow. We are somewhat surprised at the frequent recurrence of the term "otorrhœa" in alluding to the purulent discharge from middle-ear suppuration. This term ought now to be consigned to oblivion, as it i s a legacy from the days when the pathology of this affection was not understood. The author prefers Löwenberg's method of passing the Eustachian catheter, but we should like to know what authority he has for speaking of this distinguished surgeon as "Lowenburg" (pages 44 and 216). The chapters on affections of the mastoid and on the diagnosis and treatment of cerebral complications of ear diseases are distinctly good, though necessarily brief.

The illustrations are not numerous, but there are four coloured plates and thirty-four wood-cuts which add to the value of the text.

